# The Role of Healthy Diet and Lifestyle in Centenarians

**DOI:** 10.3390/nu15194293

**Published:** 2023-10-08

**Authors:** Eduardo J. Simoes, Luiz R. Ramos

**Affiliations:** 1 Department of Biomedical Informatics, Biostatistics and Medical Epidemiology, School of Medicine, University of Missouri, Columbia, MO 65212, USA; 2 Department of Preventive Medicine, Federal University of Sao Paulo, São Paulo 04024-002, Brazil; lrramos1953@gmail.com

## 1. Lifespan, Life Expectancy, and Longevity

Life expectancy at birth (hereafter, life expectancy) and longevity are established indicators of population health [1] and the economic and social development of a country [2].

Lifespan has increased substantially since the previous century, with a person born in 2014 expecting to live an average of 25 years longer than any person born in 1900 [3,4]. By 1900, the life expectancy at birth in the United States for men and women was 46 years of age and 48 years of age, respectively. By 1950, men were expected to live until 66 years of age and women until 71 years of age. In 2014, life expectancy had increased to 76 years of age for men and 81 years of age for women. What is more remarkable is that the proportion of centenarians (age 100+) has increased more rapidly than life expectancy, with it doubling in the past twenty years [5].

The increased life expectancy and longevity since 1900 parallels an epidemiological transition with a rapid decline in the incidence of and deaths caused by infectious diseases followed by an increase in non-transmissible and chronic diseases [6,7]. Most of today’s centenarians have survived past average life expectancy because they delayed the development of illness until nearer to the age of death, thus characterizing the compression of morbidity [8].

This editorial explores the role of diet and lifestyle in the human capacity to survive past the average age of death (i.e., longevity) or, more specifically, to live until becoming a centenarian.

## 2. Key Contributors to Life Expectancy Other Than Nutrition and Lifestyle

Besides nutrition and lifestyle, many factors have contributed to increased life expectancy since the late 19th century. Socioeconomic development, especially more and better jobs and higher incomes [9,10] and associated improvements in working conditions and education, advances in medical technology [11], water and sanitation [12], and mass vaccination [13,14], and the widespread availability of antibiotics [15,16] have significantly reduced the number of deaths caused by infectious diseases, especially among infants and children, thus increasing life expectancy. Moreover, technological breakthroughs in healthcare for treating and preventing cardiovascular diseases and diabetes have also contributed to preventing early deaths and prolonging life expectancy [17,18,19]. At the age of 50, these three diseases combined account for 23.7 of the additional years of life expectancy for women and 23.5 years among men who have adopted no low-risk lifestyle factors (no smoking, a normal body weight, physical activity, moderate alcohol intake, and a higher diet quality score) [20]. The worldwide decrease in the number and extent of violent conflicts (wars) and related economic improvements have also positively impacted life expectancy [21,22].

On the other hand, many factors have negatively impacted life expectancy over the same period. Among them, the growth in cigarette smoking, poor diet, and physical inactivity have contributed to epidemic levels of cardiovascular and cancer deaths and the leveling off of the life expectancy gains of the early 20th century, particularly among men [23]. Environmental pollutants [24], automobile accidents and pollution [25], and overall injuries (drug overdoses, firearm-related deaths, motor vehicle accidents, and homicides) [26] have also adversely impacted life expectancy.

## 3. How Much of Nurture and Nature Are in the Formula of Longevity?

The biology of centenarians, described in this review, provides us with clues for intervention to promote healthy ageing in the general population. One of the major reasons for this healthy ageing has to do with the genetic signature that is specific to centenarians and certainly different from octogenarians who do not enjoy the extraordinary qualities of centenarians [27].

Researchers have identified fifty genes with the potential for regulating gene processes and lifespan, with strong evidence for the critical role of DNA damage in life expectancy [28]. The contribution of genes to life expectancy has been roughly estimated to be between 20% and 40% [29]. This is in part due to their role in metabolic profiles, including insulin sensitivity and healthier levels of blood circulating lipids as well as inherited immunity profile [30,31].

Besides the contribution of genes (20–40%), diet and lifestyle appear to add substantially to longevity and the lasting functionality of centenarians.

## 4. The Role of Healthy Diet and Lifestyle in Centenarians

We postulate that a healthier diet and other lifestyle factors (i.e., no smoking, moderate alcohol intake, physical activity, social networking, and good mental and spiritual health) combined with a supportive socio-ecological environment may interact with genes to affect a person’s health and longevity.

The so-called blue zones—populations known to have a high prevalence of centenarians—are scattered around the globe, but they have some things in common: healthy diets, continual physical activity, either for leisure or due to the need to perform the activities of daily living, a strong social network based on cultural values, and health habits such as mindfulness and a purpose in life [32,33,34]. In a so-called “longevity island” in China, most centenarians’ lifestyles were found to be similar, with most participants following a light diet and not smoking or drinking alcohol [35]. Of the factors examined in this study, sleep satisfaction was the only factor significantly positively correlated with life satisfaction.

Diet and lifestyle are related to mortality among Chinese people aged 80 years and older [36,37]. In a 60-year retrospective and comparative study of Okinawans aged 65-plus (Japanese), Japanese Americans, and Americans, including diet, body mass index, and energy expenditure, long-term caloric restriction was associated with longevity and healthy aging [38]. The evidence is strong and accumulating that lifestyle changes, especially diet, have significantly and positively impacted the avoidance and delay of cardiovascular diseases, some forms of cancer, and diabetes [20,39]. Contrary to recommendations in the past 40 years, however, there is little evidence or evidence to the contrary that a low-fat diet may have any positive effect on cardiovascular risk [40,41,42]. Diet improvements such as diets low in calories and carbohydrate restriction have been associated with the prevention and, more recently, the reversal of diabetes (i.e., mean changes in hemoglobin A1c (HbA1c)), an underlying contributor to cardiovascular death and a major cause of death [43,44,45,46]. The experimental evidence is also strong for the improvement of high-density lipoprotein (HDL) function among high-cardiovascular risk individuals on a low-carbohydrate Mediterranean diet [47]. There is also strong evidence that the Mediterranean diet has clinically and statistically significant effects on the secondary prevention of major cardiovascular complications and death among cardiovascular patients [48]. Frequent intake of fruit and vegetables is inversely correlated with all-cause mortality but salted vegetables are directly associated [37].

Diet is the most important determinant in shaping the composition, diversity, and richness of gut microbiota [49]. Studies of the gut microbiota of centenarians have suggested a mark of longevity in the gut microbiota profile. People who live to a great age with different genetic, environmental, and cultural backgrounds must be analyzed and compared in the attempt to describe “universal” longevity dynamics, which is useful in unraveling how the gut microbial ecosystem can help in expanding human health span [50]. A review study suggests a causal role of the gut microbiome in host aging [51]. The microbiome is strongly associated with many clinical and lifestyle markers, especially diet and physical activity [52].

The scientific literature also underlines the positive effects of exercise on the physical capacity and longevity of incredibly old people such as centenarians [53]. Undertaking physical activity has been found to be associated with a 27% lower risk of mortality as compared with no physical activity and increased median age at death by 1.18 years [37].

There is evidence that smoking is incompatible with successful aging and compromises life expectancy even in extreme longevity [54]. Among male centenarians in China, there are associations of cognitive impairment with habits of former/current smoking and current exercise, as well as indefinite associations with habits of alcohol and tea consumption. Smoking may have a significant negative impact on cognitive function, but an ongoing exercise regime significantly improves cognitive function [55].

Finally, a greater purpose in life is associated with a reduced risk of all-cause mortality among community-dwelling older persons [56].

## 5. Conclusions

Almost every individual over the age of 60 is affected by a chronic disease/condition, from a simple cataract to complex cardiac problems. Thus, the question is, how can we determine who the healthy ones actually are? In a recent study, “centenarians presented, in general, lower morbidity and treatment burden and lower use of both primary and hospital healthcare services than octogenarians and nonagenarians, suggesting a better health status” [57]. The WHO has put forward the concept of intrinsic capacity as a composite measure of health, in which chronic conditions and body mass affect the vitality dimension, but functionality, in terms of physical and mental independence, is foremost [58,59,60]. In this scenario, the health system needs to not only exert control of all known chronic conditions using the available knowledge but also invest in disability prevention and rehabilitation strategies, based on innovation and technology.

In an ageing population, with increasing numbers of centenarians and a growing prevalence of non-communicable chronic diseases, health promotion will need to rely on encouraging individuals to adopt healthy habits such as exercise and meditation, a balanced diet, and social interaction [61,62]. From a medical viewpoint, centenarians do not escape physiological decline or age-related diseases or syndromes (e.g., frailty), but the rate of such processes is slow enough to be counterbalanced by their increased intrinsic capacity to respond to the minor stresses of daily life (i.e., resilience) [27].

In our short editorial review of the role of diet and lifestyle change in centenarians, we explored the mix of factors that contribute to longer life expectancy and longevity. We found strong evidence for an additional role of both diet and lifestyle changes as agents for longer life and reaching centenarian status. In this Special Issue, we expect researchers to add to this knowledge about increased life expectancy and longevity through studies of centenarians, including original research and synthesis on the role of diet and lifestyle changes.
